# Editorial: Non-coding RNA in diabetes and cardiovascular diseases

**DOI:** 10.3389/fendo.2023.1149857

**Published:** 2023-02-06

**Authors:** Mirjana T. Macvanin, Sonja Zafirovic, Milan Obradovic, Esma R. Isenovic

**Affiliations:** Department of Radiobiology and Molecular Genetics, VINČA Institute of Nuclear Sciences - National Institute of the Republic of Serbia, University of Belgrade, Belgrade, Serbia

**Keywords:** non-coding RNA, diabetes, cardiovascular diseases, microRNAs, long non-coding RNAs, circular RNAs, Non-coding RNA as a therapeutic target

Diabetes mellitus (DM) is a wide cluster of metabolic dysfunctions characterized by hyperglycemia resulting from insulin resistance, inadequate insulin secretion, or excessive glucagon secretion (1). The global diabetes prevalence in 2021 is estimated to be 10.5% (536.6 million people) (Li et al.). Cardiovascular disease (CVD) commonly accompanies DM and represents a leading cause of morbidity and mortality worldwide (2). An increasing body of evidence demonstrates the pivotal role of non-coding RNAs (ncRNAs) in the initiation and progression of DM and CVD (3, 4). The ncRNAs represent a group of diverse molecules that are shown to regulate gene expression and have a crucial role in physiological processes such as human growth and development, cell proliferation, apoptosis, and metabolism (5). The major classes of ncRNAs are microRNAs (miRNAs), long non-coding RNAs (lncRNAs) and circular RNAs (CircRNAs). Because of their role in maintaining physiological homeostasis through regulating the expression of various genes, miRNAs and lncRNAs attract considerable scientific interest as biomarkers for diagnosis and potential therapeutic targets in DM and CVD (3).

In this Research Topic, authors address the problem of the steadily increasing prevalence of DM and CVD globally by providing a comprehensive and up-to-date summary of various regulatory ncRNAs involved in these conditions. Furthermore, the authors discussed ncRNAs as prognostic biomarkers and therapeutic tools for diabetes and CVD. The topic received six papers, including five articles and one review.

The link between different miRNAs and platelet activation that leads to the development of cardiac disease is well established in DM. Also, Li et al. presented data from the Gene Expression Omnibus that indicate a close connection among target genes of differentially expressed miRNAs in various types of diabetes. However, the regulation and function of specific miRNAs in ischemic heart disease (IHD) still need to be clarified. Thus, Bielska et al. research has recently been focused on discovering circulating serum-derived miRNAs as potential biomarkers for early diagnosis and identification of IHD risk in DM patients. Using a novel technique, the nCounter platform for identification of differentially IHD-associated miRNAs expression, it was found that six miRNAs (miR-615-3p, miR-3147, miR-1224-5p, miR-5196-3p, miR-6732-3p, and miR-548b-3p) were upregulated in the serum of type 2 DM (T2DM) patients with IHD. In addition, 489 putative target genes were found in the endothelin-1 signalling pathway, glucocorticoid biosynthesis, and apelin cardiomyocyte signalling pathway. These findings suggest that circulating miRNAs play an essential role in developing IHD in T2DM patients and could be used as a biomarker for early detection. Furthermore, in the case–control study involving 326 patients with T2DM and 342 healthy controls Kong et al. exploreed the association between two polymorphisms of miRNA-143 and the risk of T2DM in the northern Chinese Han population. This study showed that rs4705342 might be a functional genetic polymorphism affecting the expression of miRNA-143. The CC genotype of rs4705342 might be a risk factor for the development of T2DM by increasing the expression of miRNA-143 in the northern Chinese Han population. However, more extensive population-based studies from different racial backgrounds should confirm these findings. Increasing evidence suggests that alternations of molecules in exosomes, including exosomal miRNAs, have an important development of DM and its complications (6, 7). Thus, using exosome composition have the potential as a diagnostic marker for DM. Tian et al. performed bioinformatics analysis to build lncRNA-related ceRNA networks by integrating lncRNA and mRNA expression data in human pancreatic islet-derived exosomes and to compare the regulatory role and functions of lncRNA in control and cytokine treatment exosomes. It was found that lncRNAs such as PVT1, LINC00960, and hsa-miR-107 may be involved in T1DM inflammation response, serving as novel biomarkers and potential targets for the diagnosis and treatment of T1DM. To identify new top-ranked hub miRNAs that may be involved in T2DM and Alzheimer’s disease (AD) Alamro et al. used extensive computational methods. The two strategies were set up to build classifier models: ranking miRNAs based on the number of T2DM differentially expressed genes (DEGs) they target and predicting and ranking miRNA using only the common DEGs between T2DM and its comorbidity. Obtained data indicate that T2DM DEGs targeted by hsa-mir-1-3p, hsa-mir-16-5p, hsa-mir-124-3p, hsa-mir-34a-5p, hsa-let-7b-5p, hsa-mir-155-5p, hsa-mir-107, hsa-mir-27a-3p, hsa-mir-129-2-3p, and hsa-mir-146a-5p have potential role in T2DM progression. Furthermore, the hsa-mir-103a-3p models achieved well scores for all datasets, particularly T2DM, while the hsa-mir-124-3p models achieved better results for the AD datasets. This is the first study to use predicted miRNAs to identify features that distinguish diseased samples (T2DM or AD) from normal ones, rather than traditional non-biology-based feature selection methods. One novel regulator of post-transcriptional gene expression represents tRNA-derived RNA fragments (tRFs) (8). Results of Zhao et al. show that high glucose (HG) treatment induced cardiomyocyte abnormalities, as evidenced by a decrease in cell viability and autophagy activation and increase in cell death and proinflammatory cytokine release. Furthermore, HG treatment resulted in differential expression of tRFs in cardiomyocytes, with four tRFs upregulated and one downregulated compared to the control group. In addition, four upregulated tRFs were primarily involved in cardiac dysfunction-related processes such as autophagy, insulin resistance, and DM complications. Furthermore, tRF-5014a, the most significantly upregulated tRF among all tested tRFs, was found to regulate the autophagy-related protein ATG5 expression negatively. Significantly, inhibiting tRF-5014a prevented autophagy inactivation, reduced cell viability, and increased cell death and proinflammatory cytokine release under HG conditions. These findings imply that tRFs may play a role in HG-induced cardiomyocyte injury during the progression of DM.

The Research Topic summarizes current knowledge and focuses on new insights into the effects of ncRNAs in DM and CVD. The recent findings of studies published on this Research topic, which used novel, high-throughput methodologies to identify ncRNAs involved in diabetes and CVD, necessitate a systematic approach to reviewing and summarizing their roles and potential diagnostic and therapeutic applications. However, further research is required into the molecular mechanism and its underlying role.

## Author contributions

All authors listed have made a substantial, direct, and intellectual contribution to the work and approved it for publication.
